# [Retracted] MicroRNA‑671‑5p inhibits cell proliferation, migration and invasion in non‑small cell lung cancer by targeting MFAP3L

**DOI:** 10.3892/mmr.2024.13225

**Published:** 2024-04-15

**Authors:** Junhui Ye, Wujun Luo, Lingling Luo, Limin Zhai, Pingping Huang

Mol Med Rep 25: 30, 2022; DOI: 10.3892/mmr.2021.12546

Following the publication of this paper, it was drawn to the Editors’ attention by a concerned reader that the western blotting data shown in Fig. 4B and the Transwell cell invasion data shown in Figs. 2D and 4E were strikingly similar to data appearing in different form in other articles written by different authors at different research institutes that had already been published elsewhere prior to the submission of this paper to *Molecular Medicine Reports* (one of which has been retracted). Moreover, there appeared to be inappropriately edited western blot bands featured in Figs. 4 and 5. In view of the fact that certain of the abovementioned data had already apparently been published previously, the Editor of *Molecular Medicine Reports* has decided that this paper should be retracted from the Journal. After having been in contact with the authors, they agreed with the decision to retract the paper. The Editor apologizes to the readership for any inconvenience caused.

